# Clinical Outcomes of MLC601 (NeuroAiD^TM^) in Traumatic Brain Injury: A Pilot Study

**DOI:** 10.3390/brainsci10020060

**Published:** 2020-01-21

**Authors:** Asra Al Fauzi, Krisna Tsaniadi Prihastomo, I. G. M. Aswin R. Ranuh, Tedy Apriawan, Joni Wahyuhadi, M. Arifin Parenrengi, Agus Turchan, Abdul Hafid Bajamal, Hari Basuki Notobroto

**Affiliations:** 1 Department of Neurosurgery, Faculty of Medicine, Universitas Airlangga—Dr. Soetomo General Academic Hospital, Surabaya Neuroscience Institute, Surabaya 60286, Indonesia; aswinranuh@rocketmail.com (I.G.M.A.R.R.); drtedyapri@gmail.com (T.A.); joni.wahyuhadi@gmail.com (J.W.); arifin_ns@yahoo.com (M.A.P.); agusturchan@yahoo.com (A.T.); hfbajamal@gmail.com (A.H.B.); 2 Department of Neurosurgery, Dr. Kariadi Hospital Medical Center, Faculty of Medicine, Diponegoro University, Semarang 50111, Indonesia; tsaniadi@gmail.com; 3 Department of Biostatistics and Population Studies, Faculty of Public Health, Universitas Airlangga, Surabaya 60111, Indonesia; haribasuki.n@fkm.unair.ac.id

**Keywords:** MLC601, traumatic brain injury, nonsurgical lesion, clinical outcome

## Abstract

Background: MLC601 is a natural product formulation from Chinese medicine that is extensively studied in ischemic stroke. Traumatic brain injury (TBI) shares pathophysiological mechanisms with ischemic stroke, yet there are few studies on the use of MLC601 in treating TBI. This Indonesian pilot study aimed to investigate clinical outcomes of MLC601 for TBI. Methods: This randomized controlled trial included subjects with nonsurgical moderate TBI allocated into two groups: with and without MLC601 over three months in addition to standard TBI treatment. Clinical outcomes were measured by the Glasgow Outcome Scale (GOS) and Barthel Index (BI) observed upon discharge and at months (M) 3 and 6. Results: Thirty-two subjects were included. The MLC601 group (*n* = 16) had higher GOS than the control group (*n* = 16) at all observation timepoints, though these differences were not statistically significant (*p* = 0.151). The BI values indicated a significant improvement for the MLC601 group compared to the control group at M3 (47.5 vs. 35.0; *p* = 0.014) and at M6 (67.5 vs. 57.5; *p* = 0.055). No adverse effects were associated with MLC601 treatment. Conclusion: In this cohort of nonsurgical moderate TBI subjects, MLC601 showed potential for a positive effect on clinical outcome with no adverse effects.

## 1. Introduction

Head injury is the leading cause of mortality among trauma patients [1]. Managing the sequelae of head injury remains challenging. Every year, about 1.4 million people suffer from traumatic brain injury (TBI), of which 50,000 die. More than 5.3 million Americans, or 3% of the population, live with TBI-related disability. Head injury and its consequences remain a significant global public health concern [2].

Optimal management of TBI should aim to prevent and treat the increase in intracranial pressure and other secondary brain insults, as well as to preserve cerebral perfusion pressure and optimize cerebral oxygenation. For each unique case, surgical or nonsurgical management might be the best therapeutic strategy. The treating physician must make decisions carefully to achieve good patient outcomes.

Whether the physician decides on surgical or nonsurgical management, the effectiveness of both treatments is still questionable. Despite recent advances in neurosurgical and neuro-intensive care, the long-term disabilities secondary to brain injury are still dramatic [3].

MLC601 (NeuroAiD^TM^) is an example of a pharmacological intervention for neuroprotection and neurorepair in acute ischemic stroke [4]. MLC601 (0.4 g per capsule) consists of nine herbal components (0.57 g Radix *Astragali*, 0.114 g Radix *Salvia miltiorrhizae*, 0.114 g Radix *Paeoniae rubra*, 0.114 g *Rhizoma chuanxiong*, 0.114 g Radix *Angelicae sinensis*, 0.114 g *Prunus persica*, 0.114 g *Carthamus tinctorius*, 0.114 g Radix *Polygalae*, and 0.114 g *Rhizoma acori tatarinowii*) and five animal components (0.0285 g *Cornu saigae tataricae*, 0.095 g *Buthus martensii*, 0.0665 *Hirudo*, 0.0665 g *Eupolyphaga seu steleophaga*, and 0.0285 g *Calculus bovis artifactus*). The mixture contains molecules which activate neurological repair mechanisms, including astragaloside IV, salvianolic acid B, and tanshinone IIB. MLC601 has been found to stimulate expression of brain-derived neurotrophic factor (BDNF) to enhance neurogenesis, to promote cell proliferation, and to stimulate neurite outgrowth, the development of a dense axonal and dendritic network, and to activate K_ATP_ channels [5,6]. MLC601 has been shown to significantly attenuate neurological motor deficits, brain apoptosis, and activated microglia (e.g., microgliosis, amoeboid microglia, and microglial overexpression of TNF-α) associated with cerebral contusion caused by TBI [7].

This natural product formulation has an excellent clinical and biological safety profile [8,9]. It has been studied and used widely for post-stroke recovery, showing persistent clinical benefits and safety in a large multicenter, randomized, double-blind, placebo-controlled trial. The administration of MLC601 for three months, in addition to standard stroke therapy, significantly improved the odds of achieving functional independence (modified Rankin scale from 0 to 1) compared to placebo, with significant benefit starting at six months and persisting up to 18 months after stroke [10].

Its neuroprotective effects suggest its potential to be more widely applied, for example, in TBI subjects. Theadom et al. conducted a pilot randomized, placebo-controlled clinical trial in 2018 which demonstrated beneficial effects of NeuroAiD™ on cognitive outcomes after mild or moderate TBI, particularly for complex attention and executive functioning [11]. Clinical outcomes from exploring NeuroAiD™ use among moderate TBI subjects, managed without surgery, have not been investigated.

We thus planned this pilot comparative study of NeuroAiD™, entitled “NEurological Prognosis after Brain Trauma and Use of NEuroAiD™”, or the NEPTUNE Study, to assess its effects on the functional and neurological outcome of moderate TBI subjects with nonsurgical management. The study hypothesis is that NeuroAiD™ would improve clinical outcomes in TBI as assessed by the Glasgow Outcome Scale (GOS) and Barthel Index (BI).

## 2. Materials and Methods 

This pilot study was a randomized controlled trial enrolling subjects with moderate TBI. The sample size for this study was calculated using the sample size formula for two independent samples study. This formula was designed for a study of two subject groups receiving different treatments with the primary outcome presented descriptively as median results [12].

The randomization was performed by randomly choosing a sealed envelope containing a card labeled 1 or 2 for each subject. Subjects receiving card 1 were designated to receive the additional treatment of MLC601; the subjects receiving card 2 were designated as the control group without additional treatment of MLC601. Comparisons were neither double-blind nor placebo-controlled. Administration of MLC601 was the independent variable, while Glasgow Outcome Scale (GOS) and Barthel Index (BI) were the dependent variables.

This study was performed by observing and comparing the clinical outcome of nonsurgical cases with moderate TBI who received MLC601 (treatment group) to subjects who did not receive MLC601 (control group). Written informed consent was gathered from all subjects.

We included TBI subjects with the following criteria:

### 2.1. Inclusion Criteria

- Moderate TBI (Glasgow Coma Scale or GCS of 9 to 13)
- Age 18 to 60 years old
- Closed head injury occurred within two days of MLC601 administration
- TBI with conservative/nonsurgical management
- Medically stable with no major systemic comorbidities or metabolic abnormalities
- Written informed consent

### 2.2. Exclusion Criteria

- Intracranial lesion on initial imaging or during observation for which surgery is indicated
- Bilateral unresponsive pupil
- Spinal cord injury
- History of cardiopulmonary arrest during treatment
- Life-threatening status

Standard treatment was obtained from Dr. Soetomo General Academic Hospital guidelines for TBI. Airway, breathing, and circulation stabilization were ensured in all subjects followed by symptomatic medical treatment required for each subject. Every subject received standard care and medication using the Moderate TBI Algorithm from Dr. Soetomo General Academic Hospital. MLC601 was administered via a nasogastric tube throughout the treatment duration. Treatment with MLC601 started on Day 2 in the observation ward, then continued for three months. The dose schedule was four capsules three times a day according to the standard dose of MLC601 in adults.

GOS has been widely implemented in assessing TBI outcome particularly in the first year after trauma. GOS is favored for its simplicity and due to clinicians’ familiarity with the scale. GOS has five ordinal classifications: GOS 1 is dead, GOS 2 is persistently vegetative, GOS 3 is severely disabled, GOS 4 is moderately disabled, and GOS 5 is good recovery [13].

BI is a standardized performance assessment of activities of daily living (ADLs). BI includes 10 activities to measure the need for assistance: feeding, showering, grooming, dressing, bowels, bladder, toilet use, transfers, mobility, and stairs.

Outcomes measured using GOS and BI were assessed in binomial classification as improved or not improved. Improvement was defined by the change of GOS and BI scale prior to the MLC601 administration compared to post-administration.

GOS and BI were both observed for a total of six months after discharge. Follow-up assessments were performed at months 3 (M3) and 6 (M6) through clinic visit consultations. Occurrence of any adverse effects in both groups was reported. 

Data were presented descriptively as median, standard deviation (SD), median, interquartile deviation (IQD), and minimum and maximum score. The data analysis and comparisons were analyzed using SPSS for Mac v23.0. Inferential analysis was performed using the Wilcoxon–Mann–Whitney test for comparing the two groups, and the Friedman test for comparing the times of observation in each group.

The study was conducted in accordance with the Declaration of Helsinki, and the protocol was approved by the Ethical Committee in Health Research of the Dr. Soetomo General Academic Hospital, Surabaya (0619/KEPK/Ix/2018), on 13 September 2018.

## 3. Results

### 3.1. Baseline Characteristics

A total of 32 subjects were enrolled. Table 1 shows the baseline characteristics for both groups, each having 16 subjects. The mean (SD) age was 41.9 (21.6) years in the control group and 35 (16.7) years in the MLC601 group; this difference was not statistically significant. The features and extent of intracranial lesions did not reveal any significant differences between groups. These characteristics exhibit a normal data distribution assessed and ascertained using the Kolmogorov–Smirnov test. All subjects included in this study were diagnosed with moderate TBI, where the mean (SD) of initial GCS was 10.0 (1.0) in the control group and 9.5 (1.0) in the MLC601 group.

### 3.2. The Difference in GOS between Both Groups

At discharge, median GOS in the MLC601 group was slightly higher than in the control group (3 and 2.5, respectively), as shown in in Table 2. There was no significant difference (*p* = 0.151).

**Figure 1:**
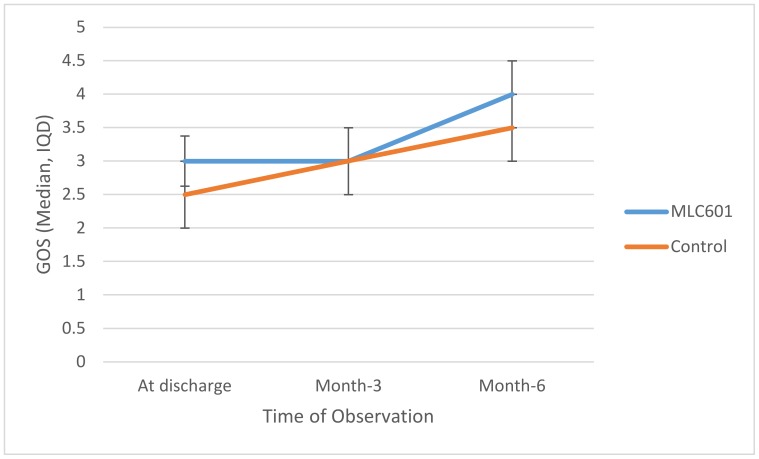
Trajectories of GOS over time between groups.

Trajectories of GOS over time are shown in Figure 1. Higher GOS was seen in the MLC601 group again at M6 compared to the control group, but this was not statistically significant (*p* = 0.354). There was a significant improvement as measured by GOS in the MLC601 group from time of discharge to M3 to M6 (3.0 to 3.0 to 4.0, respectively).

### 3.3. The Difference of Barthel Index between Both Groups

The MLC601 group had higher median BI values compared to the control group at all time points, reaching significance at M3 (47.5 vs. 35.0; *p* = 0.014) and at M6 (67.5 vs. 57.5; *p* = 0.055), as shown in Table 3.

**Figure 2:**
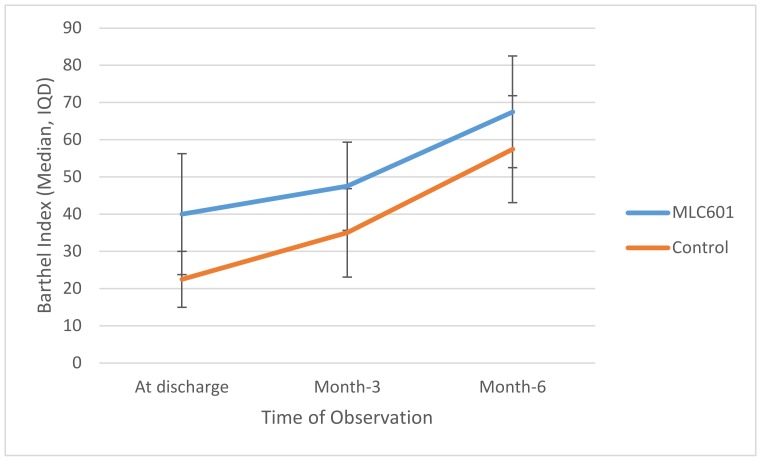
Trajectories of BI over time between groups.

Trajectories of BI over time are shown in Figure 2. There was significant improvement of BI in the MLC601 group from time of discharge to M3 to M6 (40.0 to 47.5 to 67.5, respectively).

### 3.4. Safety

None of our subjects in either group experienced any adverse effects. MLC601 was well-tolerated.

## 4. Discussion

TBI is a complex condition involving primary and secondary brain injuries. The search for combined therapies could be a better strategy for treatment, using formulations composed of more than one active ingredient. Traditional Chinese medicine (TCM) has been advocated for centuries to treat a wide variety of medical conditions. TCM consisting of multiple medicinal herbal extracts has received attention in medical academia. NeuroAiD™ has emerged as a promising treatment to support neurological recovery. Several clinical trials and reports have established its safety profile [14]. A report by Tsai et al. found that MLC601 had early positive effects in reducing TBI-induced cerebral contusions in rats. TBI-induced cerebral contusion was associated with neurological motor deficits, brain apoptosis, and activated microglia [7].

Baseline characteristics of subjects in this study, including the type of intracranial injury, did not show a statistically significant difference between the MLC601 group and the control group. Overall, these results demonstrate that the clinical outcome measured by GOS and BI was improved with the addition of NeuroAiD™ to standard nonsurgical TBI treatment. Significant improvement in BI at M3 with a positive trend close to statistical significance at M6 suggests the potential use of MLC601 in moderate TBI. The lack of significant differences in GOS may be due to the short follow-up of six months and lack of study power due to the small sample size of 32. However, the mean GOS of the MLC601 group was better than the control group at all time points. This study suggests that MLC601 may improve functional recovery and independence following moderate TBI, particularly in nonsurgical cases. 

This study evaluated the effect of NeuroAiD™ (MLC601) in moderate TBI managed conservatively without surgery. We chose to include such subjects in order to facilitate the clinical assessment of the effects of MLC601 on head trauma with minimal risk of bias.

The clinical presentation of patients with findings on CT scan suggests a focal or diffuse injury in brain tissue. The utilization of GOS and BI is appropriate for these patients as in this clinical trial. Many clinical studies try to find a better strategy to improve outcome in TBI subjects. However, trials of many neuroprotective drugs have failed to show efficacy in humans. Because of the complexity in the pathophysiology of TBI, there is increasing evidence that using combination therapies composed of more than one active ingredient could represent a better treatment strategy [7]. Interestingly, there is evidence that NeuroAiD™ acts as a neurorestorative agent targeting different pathways in the TBI cascade. This combination therapy may represent a new paradigm in the management of TBI.

Functional deficits are common neurological sequelae in TBI subjects. Recovery from brain tissue damage after TBI depends on effective stimulation of neurorepair and neuroregeneration. NeuroAiD™ (MLC601) has been shown in vitro and in animal models to demonstrate these important capacities. These mechanisms are important for activating neural plasticity and regeneration. Based on our study, subjects’ functional status (GOS and BI) during the 3–6 month observation period showed a promising improvement that indicates that MLC601 may induce neural plasticity and neuronal regeneration. Shahripour et al. also reported that MLC601 also assists functional recovery after brain infarct by increasing significantly cerebral blood flow velocity [15]. This may be mediated by an effect on stimulating microcirculation.

Clinical trials on MLC601 have not shown any serious adverse effects. Non-serious adverse effects include nausea and vomiting [16]. Ghandehari et al. [17] reported no serious adverse effects of MLC601; headache was the only non-serious adverse effect. Mild abdominal discomfort was reported by two patients after receiving MLC601. In this study, there was no report of any adverse effect after receiving MLC601.

NeuroAiD™ (MLC601) has neuroprotective and neurorestorative effects in TBI, which improve functional outcome. In this study, it has been shown that MLC601 is helpful in improving the clinical outcome of subjects with moderate brain injury who do not need surgery. This provides new input for medical treatment alternatives in addition to drugs that have been studied previously as neuroprotective and neuroregeneration agents.

As with any pilot study, ours has limitations, including the small sample size and the open label study design. However, despite such a design, we obtained a control arm with a similar pattern of baseline characteristics as those of the MLC601 group. According to a conference report from 1991, the recommended timing of outcome measurement and primary endpoint for clinical trial in moderate TBI patients (GCS 9–12) is three months [18]. A longer follow-up period might be more convincing, but this is to be balanced with the risk of increasing the number of dropouts and subject loss at follow-up.

Despite its limited number of cases, non-operative moderate TBI with cognitive impairment can become a significant burden. This study provides a novel therapy to optimize the cognitive restoration in non-operative patients. This finding is in accordance with the result of a statistically significant improvement of cognitive function as assessed by the Barthel Index.

## 5. Conclusions

This pilot study showed that MLC601 has a favorable effect in improving clinical outcomes in the setting of nonsurgical moderate TBI without any adverse effects reported during the follow-up. This is depicted in the significantly improved Barthel Index in a 3-month follow-up of the MLC601 group. In accordance with previous experimental studies on animal models and approved clinical studies, the administration of MLC601 in TBI patients showed promising outcomes in neuroprotection and neurorepair. MLC601 also assists functional recovery after brain infarct by increasing significantly cerebral blood flow velocity. Previous human clinical trials using this drug in head injuries also showed improvements in cognitive function. This pilot study is the first clinical study to examine the effects of MLC601 on moderate TBI subjects, managed without surgery. But considering the study’s limitations (small sample size, no double-blind placebo-controlled comparison, need for longer follow-up), the beneficial effects require further confirmation in larger placebo-controlled trials for TBI with longer follow-up.

## Figures and Tables

**Table 1:** Baseline characteristics.

Variables	MLC601 (*n* = 16)	Control (*n* = 16)	*p* Value
Age (Mean, SD)	35 (16.7)	41.9 (21.6)	0.320
Sex			
Male (*n* = 24)	12	12	1.000
Female (*n* = 8)	4	4	
Intracranial lesion (n, % of total per group)			
EDH	1 (6.3%)	2 (12.5%)	0.770
SDH	0 (0.0%)	1 (6.3%)	
ICH	3 (18.8%)	1 (6.3%)	
SAH	1 (6.3%)	1 (6.3%)	
Multiple lesions	7 (43.8%)	8 (50.0%)	
No lesion	4 (25%)	3 (18.8%)	
GCS (median, IQR)			
at admission	9.50 (1.0)	10.0 (1.0)	0.772

EDH = extradural hematoma; SDH = subdural hematoma; ICH = intracerebral hemorrhage; SAH = subarachnoid hemorrhage.

**Table 2:** Glasgow Outcome Scale (GOS) results.

GOS Median (IQD) Min–Max	Group		*p* *
	MLC601 (*n* = 16)	Control (*n* = 16)	
At discharge	3.0 (0.375) ^a^ 2–3	2.5 (0.5) ^a^ 2–3	0.151
Month 3	3.0 (0.5) ^b^ 2–4	3.0 (0.0) ^b^ 2–4	0.080
Month 6	4.0 (0.5) ^c^ 3–4	3.5 (0.5) ^c^ 2–4	0.354
*p* **	0.000	0.000	

* Wilcoxon–Mann–Whitney test; ** Friedman test; ^a,b,c^ the different superscripts in one column reveal differences between groups.

**Table 3:** Barthel Index (BI) results.

Barthel Index Median (IQD) Min–Max	Group		*p* *
	MLC601 (*n* = 16)	Control (*n* = 16)	
At discharge	40.0 (16.25) ^a^ 10–55	22.5 (7.5) ^a^ 10–55	0.094
Month 3	47.5 (11.875) ^b^ 25–75	35.0 (11.875) ^b^ 15–65	0.014
Month 6	67.5 (15.0) ^c^ 30–80	57.5 (14.375) ^c^ 25–75	0.055
*p* **	0.000	0.000	

* Wilcoxon–Mann–Whitney test; ** Friedman test; ^a,b,c^ the different superscripts in one column reveal differences between groups.
